# Personalized Medicine for Prostate Cancer: Is Targeting Metabolism a Reality?

**DOI:** 10.3389/fonc.2021.778761

**Published:** 2022-01-21

**Authors:** Gio Fidelito, Matthew J. Watt, Renea A. Taylor

**Affiliations:** ^1^ Department of Anatomy & Physiology, The University of Melbourne, Melbourne, VIC, Australia; ^2^ Department of Physiology, Biomedicine Discovery Institute, Cancer Program, Monash University, Melbourne, VIC, Australia; ^3^ Prostate Cancer Research Program, Cancer Research Division, Peter MacCallum Cancer Centre, Melbourne, VIC, Australia; ^4^ Sir Peter MacCallum Department of Oncology, University of Melbourne, Melbourne, VIC, Australia

**Keywords:** prostate neoplasia, lipid metabolism, obesity, metabolism, patient-derived xenograft, metabolic targeting, metabolic heterogeneity

## Abstract

Prostate cancer invokes major shifts in gene transcription and metabolic signaling to mediate alterations in nutrient acquisition and metabolic substrate selection when compared to normal tissues. Exploiting such metabolic reprogramming is proposed to enable the development of targeted therapies for prostate cancer, yet there are several challenges to overcome before this becomes a reality. Herein, we outline the role of several nutrients known to contribute to prostate tumorigenesis, including fatty acids, glucose, lactate and glutamine, and discuss the major factors contributing to variability in prostate cancer metabolism, including cellular heterogeneity, genetic drivers and mutations, as well as complexity in the tumor microenvironment. The review draws from original studies employing immortalized prostate cancer cells, as well as more complex experimental models, including animals and humans, that more accurately reflect the complexity of the *in vivo* tumor microenvironment. In synthesizing this information, we consider the feasibility and potential limitations of implementing metabolic therapies for prostate cancer management.

## Introduction

**Figure 1:**
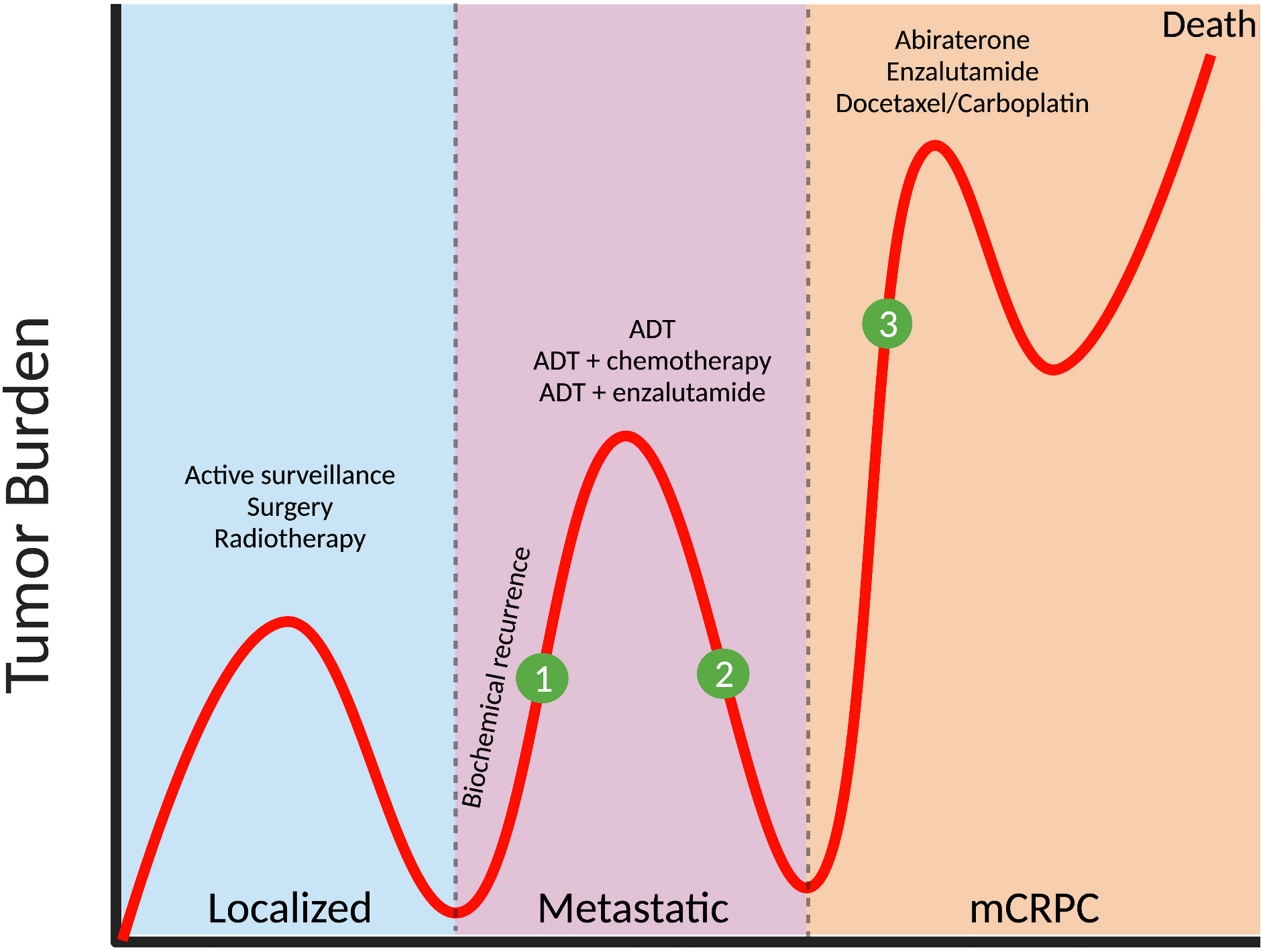
Prostate cancer progression and potential stages for intervention with metabolic therapies. The majority of patients (>80%) are diagnosed with localized prostate cancer, with treatment including active surveillance (for low-risk tumors), or surgery/radiotherapy (for intermediate- and high-risk tumors). In one third of patients, biochemical recurrence (defined as a rise in prostate specific antigen, PSA, and indicative of active tumor growth) occurs and metastases develop at distant organs, and androgen-deprivation therapy (ADT) is administered. While initially effective, tumors eventually progress to metastatic castrate-resistant prostate cancer (mCRPC) and treatments include abiraterone, enzalutamide and chemotherapy such as docetaxel or carboplatin. Clinical intervention with relevant metabolic inhibitors, that are designed to slow tumor growth, could be applied (1) at the time of biochemical recurrence, thereby delaying the need for ADT, (2) in combination with ADT to target metabolic vulnerabilities induced by androgen withdrawal or (3) to treat mCRPC in combination with, or after existing therapies.

Urological cancers accounted for 13.1% of 19.3 million new cancer incidence worldwide in 2020 (1). Prostate cancer is the most commonly diagnosed urologic cancer, followed by bladder, kidney, testis, and penile cancers (1) and frequently occurs in men over 65 years of age (2). More than 80% of men are diagnosed with localized disease, and the majority of these patients will have indolent tumors that are slow to progress, with low risk of experiencing prostate cancer-specific death (3). For these men, active surveillance, curative intent surgery or radiotherapy, are mostly effective with 10-year disease-specific survival rate of >90% (4). However, approximately one third of patients will experience disease progression and develop metastases, most commonly to bone, but also to other soft tissues such as liver and lung (5, 6). For these men, androgen deprivation therapy (ADT) is standard of care and while initially effective at reducing tumor burden, residual cancer cells adapt to low systemic androgen levels and therapy resistant metastatic castrate-resistant prostate cancer (mCRPC) develops, where tumorigenesis is driven by adaptive androgen receptor (AR) changes and intra-tumoral steroid biosynthesis (7). There are limited therapeutic options in managing this advanced stage disease, necessitating the development of novel targeted therapies and/or neo-adjuvant therapies that either prevent progression or treat mCPRC (**Figure 1**).

The hallmarks of cancer, proposed by Hanahan and Weinberg (8), comprise a series of biological capabilities acquired during the multistep development of human tumors, of which ‘deregulated cellular energetics’ is one. Cancer invokes an increase in energy production to sustain proliferation, and metabolic ‘rewiring’ is often invoked to maintain this requirement. Alterations in metabolic reprogramming include adaptation in nutrient acquisition, preferential utilization of substrates, and transcriptional changes that alter intracellular metabolic signaling pathways. Exploiting such metabolic reprogramming is proposed to enable the development of targeted therapies in cancers (9), leading to an explosion of interest in the field of cancer metabolism.

Metabolic inhibitors have been used for cancer therapies for many years, including the anti-metabolite class of chemotherapy (10, 11), and other agents have been developed for the treatment of advanced breast cancer, colorectal cancer, and hematological malignancies (12). This firmly establishes the principle that metabolic vulnerabilities can be effectively targeted for cancer treatment. However, to date, there are no metabolic inhibitors approved for use in prostate cancer, which we posit is due to a knowledge gap in understanding the molecular and cellular reprogramming and associated changes in substrate utilization in human tumors, and the marked heterogeneity of this disease.

Herein, we will discuss how metabolism is reprogrammed in prostate cancer, in both localized and mCRPC, which likely have different metabolic needs. We will focus on literature employing studies in immortalized prostate cancer cells and expand to more complex environments, including animal models and human studies. We will then outline the factors contributing to variability in prostate cancer metabolism, including genetic drivers and alterations in the tumor microenvironment (TME), and lastly discuss the feasibility of metabolic targeting in patients and potential limitations in prostate cancer management.

## Prostate Cancer Metabolism

**Figure 2:**
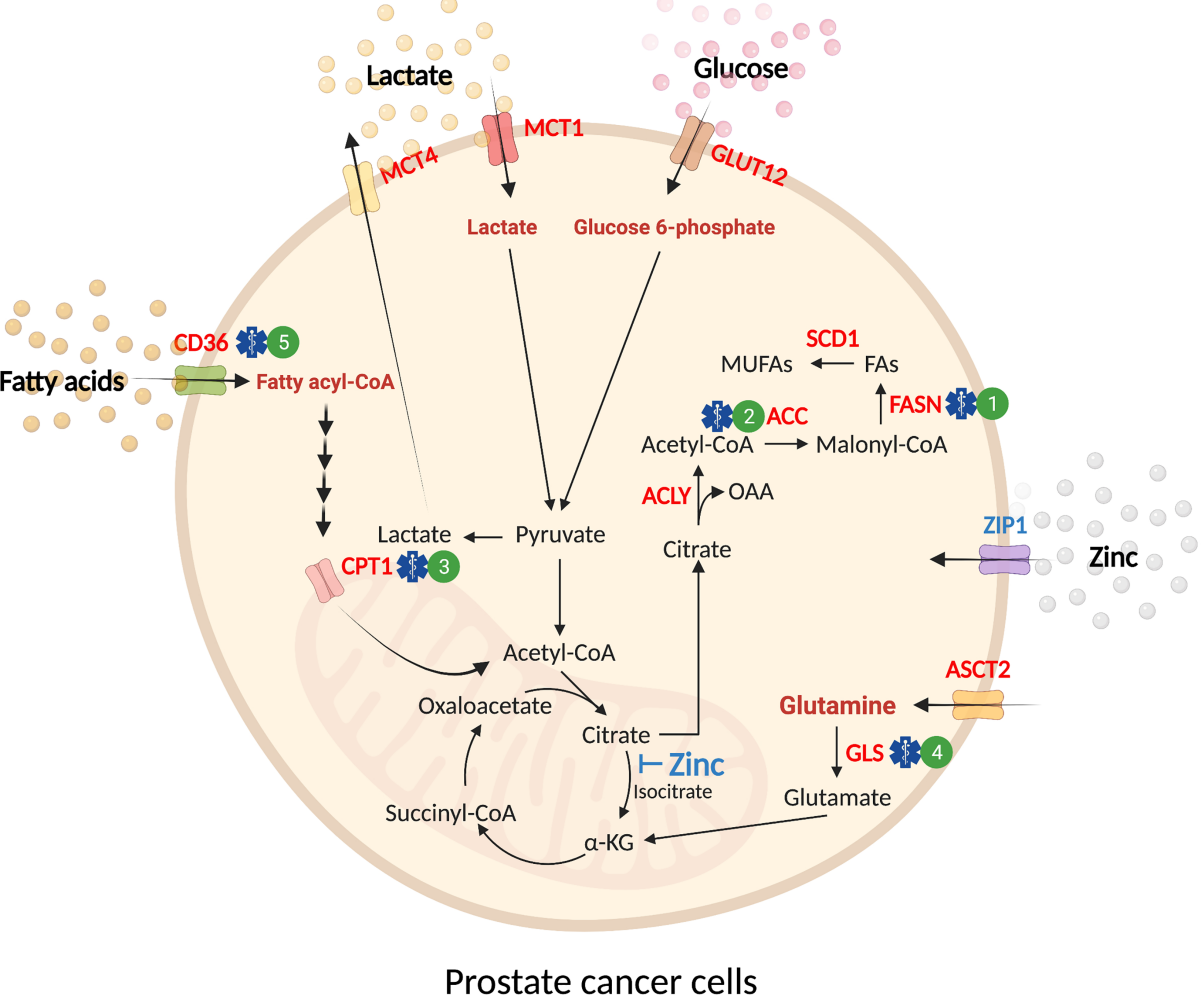
Substrate utilization in prostate cancer. Normal prostate epithelial cells exhibit a glycolytic phenotype due to the inhibitory effect of mitochondrial zinc accumulation in the TCA cycle (*blue text*). Malignant transformation of prostate epithelial cells leads to an increase in the uptake of exogenous nutrients (glucose, glutamine, fatty acids, and lactate) and *de novo* synthesis of lipids (*red text*). These substrates are utilized for energy production in the mitochondria to accommodate increasing energy demands in malignancy. In prostate cancer, glucose uptake is mediated by GLUT12 before it is catabolized into pyruvate. While a proportion of glucose-derived pyruvate enters the TCA cycle for oxidation, a fraction of pyruvate is reduced to lactate and transported out of the cell by MCT4. The influx of extracellular lactate is mediated by MCT1. In mitochondria, the outflow of citrate to cytosol provides substrate for *de novo* synthesis of fatty acids (i.e. lipogenesis). ASCT2 supplies exogenous glutamine as a fuel source through deamination by glutaminase (GLS) before further conversion into ⍺-KG to feed the TCA cycle. Fatty acid uptake is mediated by fatty acid translocase (FAT)/CD36 before transport into the mitochondria by CPT1. In mitochondria, fatty acids undergo β-oxidation, producing acetyl-CoA that feeds into the TCA cycle. Pre-clinical treatments for prostate cancer are denoted by a blue symbol and corresponding number. 1) FASN (*e.g.* TVB-2640, IPI-9119); 2) ACC (*e.g.* Firsocostat, PF-05175157); 3) CPT1 (*e.g.* Perhexiline); 4) GLS (*e.g.* CB-839); 5) CD36 (*e.g.* agents in development). ⍺-KG, ⍺-ketoglutarate; CPT1, carnitine palmitoyltransferase 1; FAs, fatty acids; GLS, glutaminase; MCT1, monocarboxylate transporter 1; MCT4, monocarboxylate transporter 4; MUFAs, monounsaturated fatty acids; OAA, oxaloacetate.

The prostate gland secretes large amounts of citrate (~1000-fold than blood plasma) as the major constituent of prostatic fluid (13). The accumulation of zinc within the prostate gland by ZIP1 (SLC39A1) competitively inhibits mitochondrial aconitase (ACO2) activity, which hinders citrate oxidation and Tricarboxylic Acid (TCA) cycle flux (14–16). Hence, unlike other well-differentiated tissues, which rely on oxidative phosphorylation to produce ATP, normal prostate epithelium depends on aerobic glycolysis with glucose and aspartate as the primary carbon donors (17). In malignant prostate tissues, ZIP1 expression and citrate production are decreased, while ACO2 expression is increased, converting prostate cells from citrate‐producing to a citrate‐oxidizing phenotype (18–22). These changes enhance the capacity for energy production to support proliferation and metastasis, and provide evidence that metabolic adaptation occurs in prostate cancer (**Figure 2**).

### Glucose

Glucose is a primary substrate for most cells. Glucose is transported into cells and undergoes glycolysis, resulting in the production of pyruvate. A proportion of pyruvate undergoes reduction to lactate, but the majority enters the mitochondria for further processing in the TCA cycle for eventual oxidative phosphorylation, which enables energy production. The glycolytic intermediates generated in the breakdown of glucose are also used for nucleotide, amino acid, and lipid biosynthesis (23). In contrast, most cancer cells utilize glucose differently, producing lactate at high rates despite the presence of oxygen in a phenomenon termed Warburg metabolism or aerobic glycolysis (24).

Glucose utilization in increased in prostate cancer compared to normal tissues, and actively contributes to the growth of prostate cancer cells. Treatment of AR-positive LNCaP prostate cancer cells with the synthetic steroid R1881 induced transcriptional upregulation of glucose transporters (GLUT1, GLUT12) and glycolytic enzymes (HK1/2, and PFKB2), increased glucose uptake, glucose entry into glycolysis, and glucose storage into lipids (*de novo lipogenesis*) (25–27). Meanwhile, studies conducted in AR-negative cells (PC3 and DU145) reported higher glucose uptake and increased lactate production compared to AR-positive cells (LNCaP and 22Rv1) (28–30). These observations indicate that AR signaling promotes the entry of glucose-derived pyruvate into the TCA cycle for eventual complete oxidation, while aerobic glycolysis is increased in the absence of AR signaling in immortalized prostate cancer cells.

Enhanced aerobic glycolysis in response to androgen withdrawal is also observed in *in vivo* models. A metabolomic screen of an orthotopic xenograft model of TRAMP-C1 prostate cancer demonstrated increased glycolysis in tumors following androgen deprivation (31) while, *in vivo* and *ex vivo* metabolic imaging using hyperpolarized 1-[^13^C]pyruvate in TRAMP tumors also points towards elevated glycolysis and higher lactate dehydrogenase (LDH) activity in the castrate setting (30).

Evidence from human studies similarly show different glucose utilization across the spectrum of prostate cancer, which is best illustrated by ^18^F-fluorodeoxyglucose (^18^F-FDG) cancer diagnostic imaging in patients. ^18^F-FDG is taken up by tissues and ‘trapped’, and its accumulation is reflective of the tissues glycolytic activity (32). Notably, the diagnostic utility of ^18^F-FDG imaging is limited to localized high-risk tumors and metastatic disease, indicating increased glucose uptake in rapidly growing malignant tissues and not indolent localized disease [*as reviewed in* (33)]. In addition, proteins that regulate glucose metabolism were increased in both localized and metastatic lesions of prostate cancer, including HIF-1⍺, GLUT1, HK2, PFKFB3, PFKFB4, PKM2, PDK1 (29, 34–37). Functional analysis of glucose metabolism showed increased *de novo* lipogenesis in localized prostate cancer tissues compared to patient-matched benign tissues (38); however, this was not accompanied by increased glucose oxidation, indicating that much of the additional citrate produced in the TCA cycle is exported into the cytosol for lipogenesis (38).

Lactate is produced *via* the reduction of pyruvate and is classically viewed as the by-product of excess glycolysis; however, it is becoming increasingly recognized as an important mediator of tumorigenesis in some cancers. Lactate is used to fuel the TCA cycle in some malignancies (*e.g.*, non-small cell lung cancer) (39) and inhibiting lactate influx into cells through the monocarboxylate transporter 1 (MCT1) reduces the metastatic potential of melanoma (40). Serum lactate dehydrogenase (LDH) is often increased in patients with high-grade prostate cancer and is associated with increased risk of mortality and disease progression in patients with metastatic prostate cancer (41, 42). Consistent with these observations, clinical studies utilizing hyperpolarized ^13^C-pyruvate imaging reported a positive correlation between prostate cancer Gleason grade and the conversion of pyruvate to lactate (43). Interestingly, monocarboxylate transporter 4 (MCT4), the protein responsible for lactate efflux from cells, is increased in localized and metastatic tumors (29) and RNAi-mediated silencing of MCT1/4 in prostate cancer cells decreased cell growth (44), suggesting lactate production and its intracellular utilization are important for tumorigenesis. A more comprehensive investigation of lactate metabolism in prostate cancer is clearly warranted. Finally, the pentose phosphate pathway (PPP) is a glucose catabolic pathway that appears to be important in prostate tumor growth in AR/SREBP/6PGD-dependent manner (45). However, whether PPP plays a significant role in prostate cancer by generating nucleotide precursor or sustaining the NADPH pool for lipogenesis and redox homeostasis is yet to be elucidated.

### Glutamine

Glutamine is a nonessential amino acid and the most abundant amino acid in the circulation (~500 µM). Glutamine functions as a carbon donor for lipogenesis *via* reductive carboxylation, a nitrogen donor for non-essential amino acid production and nucleotide biosynthesis (46), and as a fuel source (47–51). Glutamine anaplerosis starts with glutamine conversion into glutamate by glutaminase (GLS) then further conversion into ⍺-ketoglutarate (AKG) to feed the TCA cycle by the actions of glutamate dehydrogenase (GLUD) and several transaminases, including glutamate–oxaloacetate transaminase (GOT), glutamate–pyruvate transaminase (GPT), and phosphoserine transaminase (PSAT) (46). While fourteen proteins are known to transport extracellular glutamine into cells, SLC1A5/ASCT2 is thought to be the major transporter, and its expression is upregulated in various cancers (52, 53). Glutamine can also donate its alpha nitrogen to serine, glycine, alanine, or aspartate following deamidation to glutamate (54). Serine feeds into one-carbon metabolism, which centrally integrates many pathways that are dysregulated within prostate cancer, strengthening the argument for targeting glutamine metabolism (55, 56). Additionally, enhanced aspartate metabolism has been implicated with epithelial to mesenchymal transition while increased levels of alanine has been identified within prostate cancer biopsies (57, 58).

Several lines of evidence demonstrate an important role for glutamine in prostate cancer growth and progression. ASCT2 is expressed in prostate cancer cells (*e.g.*, LNCaP, VCaP, PC3, and DU145) (53, 59, 60) and approaches that reduce ASCT2 expression/function suppress glutamine uptake and hamper cell proliferation (53, 59). In a similar manner, GLS expression is higher in prostate cancer cells (e.g., LNCaP, 22Rv1, DU145, and PC-3) as compared with non-malignant prostate epithelial cells (e.g., RWPE-1) (60–62), and selective inhibition of GLS reduced proliferation and survival (60–63).

Key findings in cultured cells have been recapitulated in mouse models. ASCT2 mRNA expression is decreased upon castration and increased in CRPC (59) and knockdown of ASCT2 suppresses growth and metastatic burden in PC3 xenografts in mice (59), although rates of glutamine uptake and downstream metabolism were not assessed in this study. GLS expression is increased post-castration in LNCaP and LAPC4 xenografts (61), and pharmacological inhibition of GLS1 reduces the tumor burden in PC3, but not LNCaP xenografts (61), highlighting the dependency on glutamine metabolism in AR-negative, hormone-insensitive prostate cancer (61). Consistent with this notion, analysis of TRAMP tumors utilizing [U-^13^C] glutamine metabolic tracing reported upregulation of glutaminolysis to replenish TCA cycle intermediates and upregulation of GLS1 activity in castrate-resistant compared to androgen-dependent tumors (30).

The importance of glutamine metabolism in human prostate cancer is unknown. ASCT2 and GLS1 mRNA expression is high in human prostate cancer (59, 63, 64) and ASCT2 expression is significantly associated with shorter time to biochemical recurrence in recurrent prostate cancer (64). Temporal ASCT2 expression is also observed in human tumors, with decreased expression upon ADT treatment (1-6 months and 7-12 months) and increased expression in recurrent tumors (59). In addition, expression of the GLS1 enzyme undergoes a shift in isoform from kidney-type glutaminase (KGA) to the more active isoform, glutaminase C (GAC). This shift occurs progressively from localized to mCPRC and neuroendocrine prostate cancer (NEPC) (61). While these observations signal an important role for glutamine metabolism in advanced stages of prostate cancer (*i.e.*, mCRPC and NEPC), studies evaluating glutamine uptake, glutaminolysis and ATP production in human prostate cancer are clearly needed.

### Fatty Acids

Fatty acids are essential for the generation of structural cell membranes, energy production, and cellular signaling. Fatty acids are derived from adipose tissue lipolysis or from triglycerides stored in chylomicrons and very-low density lipoproteins, where they are transported from the circulation into cells. Several cell types, most notably hepatocytes and adipocytes, are capable of synthesizing fatty acids using other substrates, such as glucose and acetate, through a process called *de novo lipogenesis*. Fatty acids are the dominant metabolic substrate in most tissues where they undergo mitochondrial β-oxidation to generate acetyl-CoA, which feeds into the TCA cycle and oxidative metabolism.

Emerging evidence demonstrates an important role for fatty acid metabolism in prostate cancer. Fatty acid uptake is increased in immortalized prostate cancer cells (38, 65), which is often accompanied with increased energy production from fatty acid oxidation (65, 66). Treatment of prostate cancer cells with etomoxir, an inhibitor of fatty acid oxidation, reduces cell viability and proliferation, reinforcing the importance of this metabolic substrate for cancer progression (65, 67). Aside from the direct energy-generating mitochondrial fatty acid oxidation, peroxisomal fatty acid oxidation also supports prostate cancer growth (68, 69).

As mentioned above, prostate cancer is exceedingly lipogenic, highlighted by accelerated *de novo* synthesis of fatty acids driven by enhanced activity of sterol regulatory element-binding protein (SREBP) (70, 71), which induces the transcription of many genes involved in lipid metabolism, including ACLY, ACACA, FASN, SCD1 and LDLR (72). Studies employing pharmacological and genetic manipulation of key regulatory enzymes of lipid metabolism in immortalized cell lines and xenografts have demonstrated the importance of several lipid metabolism pathways in prostate cancer progression including increased *de novo* lipogenesis (i.e., *via* ACLY, ACC and FASN inhibition) (73–76), triacylglycerol storage (DGAT1) (77), cholesterol metabolism (SOAT1, HMGCS1, HMGCR, and SCARB1) (78–80), lipolysis (MAGL) (81), and fatty acid elongation (ELOVL5 and ELOVL7) (82, 83). Similarly, 2,4-dienoyl-CoA reductase (DECR1) and enoyl-CoA delta isomerase 2 (ECI2), auxiliary enzymes responsible for the degradation of unsaturated fatty acids, are also essential for prostate cancer growth and therapy resistance (84–86). Finally, studies using tandem mass spectrometry lipidomics have reported marked alterations in the prostate lipidome with cancer (38, 82, 87, 88), indicating the likelihood that other nodes of lipid metabolism are regulated in prostate cancer development and metastasis.

While studies in cells and mice provide a reasonably compelling narrative that distinguishes lipid metabolism as a hallmark of prostate cancer, studies in primary human tissue are limited. Our team recently performed functional metabolic analysis in freshly procured human prostate tissue. Fatty acid uptake, fatty acid storage into complex lipids and cellular membranes, and *de novo* lipogenesis were upregulated in malignant compared to benign prostate tissues (38). Further studies identified fatty acid translocase (FAT/CD36) as a key fatty acid transport protein in prostate cancer while inhibition of FAT/CD36 with a monoclonal antibody attenuated tumor growth in a prostate patient-derived xenograft (PDX) and PDX-derived organoids. While this study identified a role for altered lipid metabolism in localized disease, further studies are required to ascertain whether these, and other changes in lipid metabolism, occur in metastatic disease. Additionally, whether there are further alterations in fatty acid utilization in the setting of mCRPC, where AR activity is amplified, is yet to be determined. In this context, a recent study employing transcriptomics and proteomics in prostate cancer cell lines and patient samples identified several lipid-mediated transporters and increased rates of fatty acid, cholesterol, and low-density lipoprotein uptake with androgen stimulation (89). Hence, any potential therapeutic benefit is likely to require cotargeting of lipid supply.

Efforts to elucidate the metabolic landscape of prostate cancer have highlighted the importance of glucose, glutamine, and fatty acid in prostate cancer growth and progression, and it is evident that there is a ‘*metabolic switch*’ from normal prostate epithelium to prostate cancer (90). However, the differences in metabolic regulation between localized and mCRPC tumors are less well defined. This highlights the need for comprehensive studies evaluating multiple substrates in a more complex system that reflect clinical tumors. The current advancement in patient-derived organoids (PDO) generation protocols (91, 92) and the creation of several PDX collections (93–96) will enable complex studies in identifying targetable metabolic vulnerabilities in different disease stages. However, a limitation of all *in vitro* studies is that metabolite concentrations in the TME are unknown. A widely held view is that commonly used cell culture medium (*e.g.*, RPMI, MEM, DMEM) contain significantly higher concentrations of glucose and amino acids than what is physiologically available, and often do not contain free fatty acids. Acknowledgement of this limitation and a better understanding of the constituents of the TME in different disease stages is required to move the field forward (see **Table 1**).

**Table 1:** Methodological considerations.

Limitations of experimental models used to assess metabolism in prostate cancer
• Most studies assessing metabolic regulation have been conducted in immortalized prostate cancer cells, including PC3, DU145 and LNCaP, which facilitate simple physiological and/or genetic manipulation and high throughput analysis, but bare limited resemblance to the complexity or heterogeneity of human tumors.
• Exposure of cells *in vitro* to supraphysiological nutrient levels in the culture medium unlikely recapitulate the condition in tumors, although it is noted that the concentration of metabolic substrates in the tumor microenvironment (TME) are currently unknown.
• *In vivo* studies using genetically engineered mouse models of prostate cancer overcome some of these issues, however the mutations do not replicate the genomic and phenotypic heterogeneity observed clinically.
• The use of human tissues or clinical studies are often impracticable due to limited access to patients under carefully controlled conditions and the technical difficulty in assessing tissue-specific metabolism *in vivo*.
• To overcome this limitation, prostate cancer patient-derived xenografts (PDXs) capture the heterogenous nature of tumor of origin. However, despite its perceived superiority over other approaches, PDXs lack stroma and immunological contribution.
• Combined approaches that integrate these complementary models are required to understand the metabolic landscape of prostate cancer and identify promising therapeutic strategies.

## Factors Influencing Prostate Cancer Metabolism

**Figure 3:**
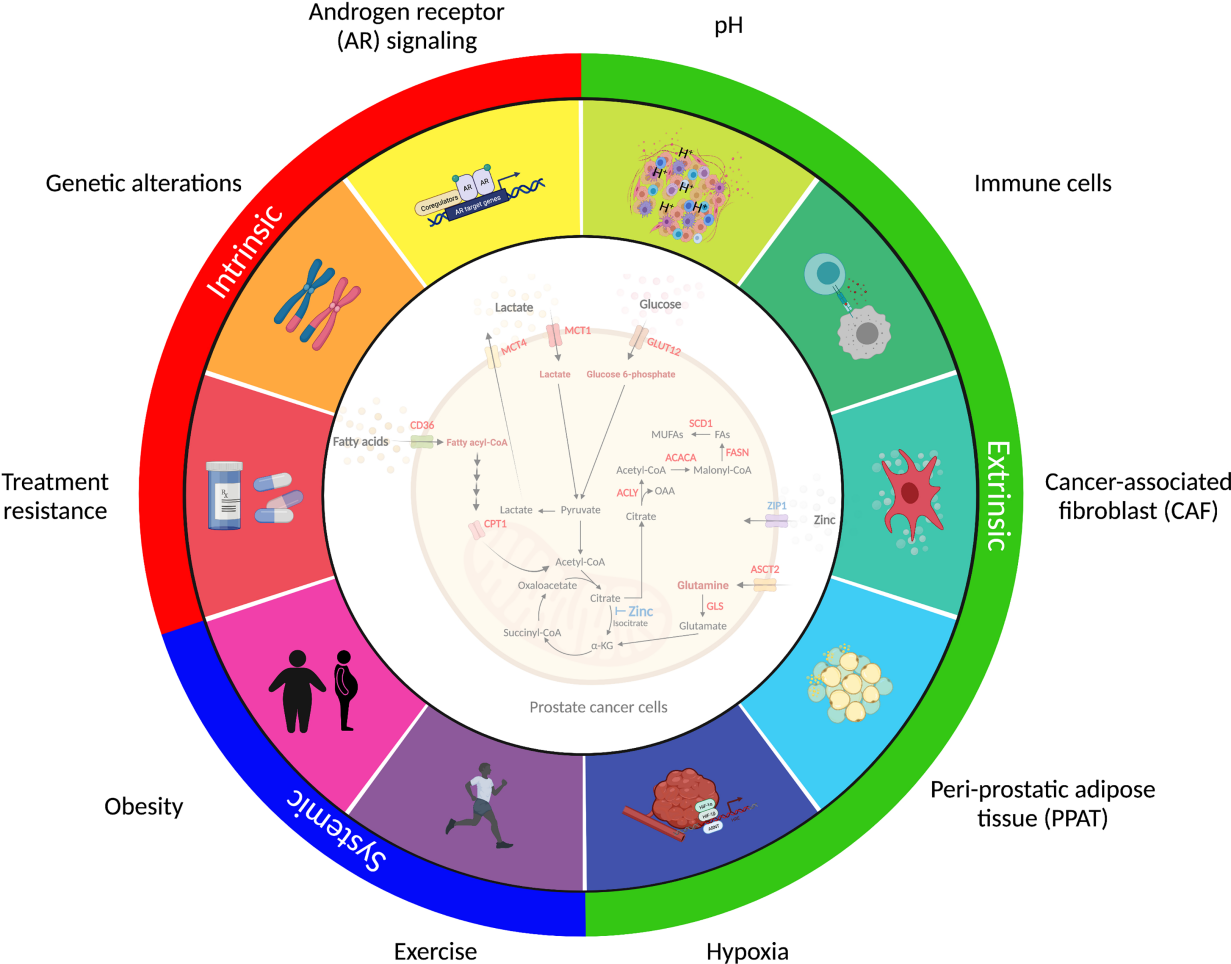
Factors shaping prostate cancer metabolism. Prostate cancer metabolism is influenced by factors inside the cancer cell (*intrinsic*), immediately adjacent to the cancer cell (*extrinsic*), and derived from multi-system perturbations (*systemic*). The intrinsic factors (*red*) represent intracellular changes regulated by androgen receptor (AR) signaling activity, genetic alterations, and therapy resistance. The extrinsic factors (*green*) are extracellular signals derived from nearby cells that influence cancer cell metabolism and adaptation. These include dysregulated vascularization, acidosis, hypoxia and cancer-associated fibroblasts (CAF), immune cells and peri-prostatic adipose tissue (PPAT), which collectively contribute to the tumor microenvironment (TME). The systemic factors (*blue*) that regulate tumor metabolism include alterations in the body’s metabolic and hormonal milieu, induced by short-term perturbations or long-term changes in metabolic state, including exercise and obesity, respectively. These complexities should be considered when assessing metabolic changes in prostate cancer and highlight the multiple challenges in implementing metabolic therapies into clinical practice.

Prostate cancer displays marked heterogeneity from a molecular, morphological and clinical perspective and consideration of the factors that influence metabolic selection is essential to better understand the metabolic requirements of human prostate tumors in their native environment (**Figure 3**).

### Genetic Drivers of Metabolism

Specific oncogenic mutations can promote metabolic phenotypes in some cancers [*as reviewed in* (97)]. This is unequivocally the case in melanoma, where *BRAF^V600E^* mutations that account for ~80% of melanomas drive a metabolic program with a preference towards Warburg metabolism (98). Inhibition of oncogenic *BRAF* using drugs such as vemurafenib, dabrafenib or encorafenib cause profound reductions in glucose uptake and improve patient outcomes (99). While the use of oncogene-driven mouse models has been helpful in linking specific genomic alterations with aberrant metabolic phenotypes in some tumor types (100–103), the profound number of molecular aberrations and heterogeneity observed in human tumors make it challenging to identify single DNA or gene alterations that dictate metabolic regulation.

Acquisition of genomic alterations underpins prostate tumorigenesis. Comprehensive genomic characterization of prostate cancer has identified recurrent alterations in genes involved in androgen signalling, DNA repair, and PI3K signalling, such as *TP53*, *SPOP*, *PTEN*, *AR*, *FOXA1*, *MYC*, *ATM* and *APC.* However, the incidence of significantly mutated genes follows a long-tail distribution, where the frequent alterations are only detected in ~5-10% of cases, and many other genes are mutated in <3% of cases (104). This underpins a complex genetic landscape in prostate cancer and heterogeneous nature of the disease. There is limited evidence showing induction of metabolic remodelling by individual oncogenes, such as *MYC* amplification, which promotes fatty acid synthesis and accelerates prostate cancer progression (105, 106). However, the absence of a dominant and frequent genetic mutation in prostate cancer indicates that a ‘common’ oncogenic-driven metabolic phenotype is unlikely to exist, although this remains to be fully explored.

### Neuroendocrine Prostate Cancer

The prominent pathology in prostate cancer is adenocarcinoma; however, in rare cases (<1%), NEPC tumors occur and present with an AR-null phenotype. While these are uncommon at diagnosis, there is increasing prevalence of therapy-induced NEPC that develops as an aggressive form of mCRPC. Treatment of NEPC presents an unmet clinical challenge in managing advanced prostate cancer. Emergence of an AR-null, NEPC phenotype is characterized by the expression of neuroendocrine markers such as synaptophysin, CD56, and chromogranin, with the absence of AR and AR-regulated gene expression (107). The genomic loss of tumor suppressors, dysregulation of specific transcription factors, and epigenetic modifications have been linked to the gain of neuroendocrine-like properties [*as reviewed in* (108)].

The metabolic regulation in NEPC disease states requires independent investigation. A previous study identified the requirement of increased serine biosynthesis following the loss of PKCλ/l to fuel the methionine salvage pathway, which in turn augmented NEPC differentiation through DNA methylation (56). This highlights the role of metabolism in epithelial cell differentiation, beyond energy production. NEPC is characterized by increased glucose uptake and glucokinase expression compared to adenocarcinoma, despite the suppression of GLUT12 (109). Transcriptomic analysis of NEPC PDX and patient specimens identified elevated glycolysis and lactate production as the metabolic feature of NEPC (110); however, these and other metabolic processes in NEPC are yet to be quantified using appropriate tracer methodologies.

### Prostate Metabolism and the Tumor Microenvironment

Prostate tumor cells reside in close proximity to neighboring cells within the tumor microenvironment (TME). The major components of the TME include cancer-associated fibroblasts (CAFs), endothelial cells, mesenchymal cells, as well as immune cells such as mast cells, T cells, macrophages and monocytes. Each of these cell types secrete metabolites, hormones, extracellular vesicles and cytokines that could impact local metabolism. Several characteristic changes in the TME can impact metabolism, including dysregulated vascularization that involves disorganized and leaky blood vessels with low pericytes coverage, which in turn creates a hypoxic and acidic environment. Hypoxia has been implicated in the metabolic reprogramming of cancer cells, and upregulation of HIF-1α plays an important role in the regulation of glycolysis (111). Additionally, CAFs themselves undergo a significant shift from oxidative phosphorylation to aerobic glycolysis, altering substrate availability for nearby cancer cells (112). There are also remarkable alterations in immune responses and the inflammatory environment in the TME, creating an immunosuppressive milieu. Interestingly, this relationship is likely to be bidirectional, with evidence that the low pH induced by excess lactate production in cancer cells reduces T cell infiltration. In addition, the microbiome is an important modulator of various host processes such as metabolism and immunity, and microbiome dysbiosis is associated with tumor development, disease progression, and treatment response and resistance in prostate cancer (113). The complexity of intra-tumoral paracrine signaling is exacerbated by remarkable heterogeneity in the cellular composition between individual human tumors, as demonstrated by single-cell transcriptomic analysis (114). These findings highlight the need to develop methods to sample and define the components of the prostate TME, with a view to understanding the factors controlling tumor metabolism and, perhaps, determining targetable metabolic vulnerabilities.

### Prostate Cancer Metabolism and Obesity

Obesity is a global epidemic affecting 281 million men (115) and more than 40% of men aged between 45-74 are obese (116), an age when the majority of prostate cancer diagnoses occur (117). While there is limited evidence that obesity is an initiator of prostate cancer (118, 119), epidemiologic evidence indicates that obese patients develop aggressive tumors with poor clinical outcomes (120, 121), although there is some conjecture with respect to mCRPC (122). Studies in rodents mostly confirm progression towards an aggressive phenotype in obesity [*as reviewed in* (123)] and many plausible mechanisms have been proposed to explain the link between obesity and aggressive prostate cancer (123), including increased free fatty acid supply, hyperinsulinemia, hypertriglyceridemia, altered endocrine signaling and low-grade inflammation (123). Notably, definitive evidence supporting these putative obesity-related drivers of prostate cancer progression is lacking. While not directly related to obesity, higher dietary saturated fat intake is associated with prostate cancer lethality (105) and raises the possibility that dietary interventions that reduce saturated fat intake and/or interventions to desaturate fatty acids might be efficacious in managing prostate cancer.

Periprostatic adipose tissue (PPAT) covers the prostate anteriorly and patients with more PPAT have worse cancer prognosis (124), leading to the view that PPAT secreted factors stimulate tumorigenesis, particularly in obesity (123). Studies employing co-culture of prostate cancer cell lines and adipocytes (125), or the addition of PPAT secreted factors to prostate cancer cells (126, 127) support this possibility; however, co-grafting of patient-matched PPAT and localized prostate cancer PDX did not enhance prostate cancer tumorigenesis in mice (126). Nevertheless, changes in fatty acid delivery or adipose-secreted proteins (*i.e.*, adipokines) from PPAT are factors that may impact prostate cancer metabolism.

### Exercise and Prostate Cancer

Observational studies indicate that exercise and physical activity are associated with decreased risk of prostate cancer incidence, and lower overall prostate cancer mortality. Notably, vigorous activity is associated with a reduced risk of advanced, high Gleason grade group, or fatal prostate cancer in men over 65 years of age (128). While the mechanisms underlying potential anti-tumorigenic effects of exercise remain elusive, several have been proposed and include reduced circulating insulin, insulin-like growth factor 1 and proinflammatory cytokines, reduced tumor vascularization, AR adaptations, reduced cholesterol, production of unknown ‘exercise circulating factors’ contained in exosomes and reprogramming of metabolic and immunological dysregulation (129, 130). Overall, local, systemic and external influences play a significant role in metabolic regulation and prostate tumorigenesis, although there remains much to be learnt in this space.

## Systemic Therapies for Prostate Cancer

Hormone therapy is standard of care for patients with advanced prostate cancer, involving the use of gonadotropin-releasing hormone (GnRH) agonists or antagonists to suppress testicular testosterone synthesis. The use of androgen-targeted agents, such as enzalutamide (AR antagonist) and abiraterone (inhibitor of cytochrome P450 (CYP) C17 to block androgen synthesis), are used clinically to treat mCRPC (131). Meanwhile, Rucaparib (132) and Olaparib (133) (PARP inhibitor) have been recently approved for men with mCRPC harboring deleterious mutation of homologous recombination repair genes. While these discoveries improve current management of prostate cancer, the need for new therapeutics or adjuvant therapies continues as mCRPC remains lethal, and NEPC tumors are refractory to hormone therapy.

Metabolic changes, including insulin resistance, dyslipidemia, diabetes, and cardiovascular morbidity have been associated with ADT (134). Recent studies examining metabolomic profiles of men receiving ADT reported a reduction in acyl-carnitines and ketone bodies, indicating ADT-induced systemic changes in fatty acid metabolism (135, 136). Meanwhile, low-carbohydrate diets reversed this alteration in fatty acid metabolism while slightly increasing androgen suppression (137). This emphasizes the importance of diet in maximizing ADT therapeutic activity while minimizing its effects on altering metabolism. In addition, patients are often prescribed with exercise, anti-hypertensive, anti-hyperlipidemic and anti-hyperglycemic medications to attenuate the effects of ADT, and it is possible that these interventions induce metabolic changes that improve cancer outcomes, although the evidence for this is limited (discussed below).

Aside from the impact of ADT on systemic metabolism, it has been postulated that ADT induces metabolic vulnerabilities in the tumor itself that can be therapeutically targeted using combination approaches. The use of metabolic inhibitor(s) as an adjuvant therapy have improved the efficacy of existing therapies and prevented the development of resistance in several tumors (138). In prostate cancer, metabolic adaptations occur in prostate cancer cells following ADT, as well as androgen-targeted therapies, including enzalutamide or abiraterone, suggesting the possibility of co-treatment strategies (139, 140) (**Figure 1**). This was exemplified in prostate cancer PDXs where a synergistic effect was demonstrated following treatment of ADT (through castration) plus metformin (141). Thus, the possibility of metabolic targeting in combination with ADT should be further explored.

## Putative Metabolic Targeting in Prostate Cancer

Effective targeting of cancer metabolism relies on suppressing or modulating metabolic pathways identified as cancer ‘dependent’ and the use of metabolic agents is thereby limited by the defined therapeutic window of efficacy and toxicity in cancerous and non-cancerous cells. While there are no metabolic inhibitors approved for clinical use in patients with prostate cancer, several agents targeting *de novo* lipogenesis, fatty acid oxidation and glutamine oxidation are in pre-clinical or early phase clinical trials.

### *De Novo* Lipogenesis Inhibitors

The lipogenic phenotype of prostate cancer raises the possibility of targeting *de novo* lipogenesis. In this context, fatty acid synthase (FASN) is a rate-limiting enzyme in this process, and several FASN inhibitors, including TVB-3166 and TVB-2640, suppressed tumor growth by 15% in 22Rv1 xenografts (142), and notably, induced up to 97% tumor growth inhibition in combination with paclitaxel (142). Another FASN inhibitor, IPI-9119, showed anti-tumorigenic activity in human mCRPC organoids and 22Rv1 and LNCaP-95 xenograft models (143). Phase I studies of TVB-2640, the first FASN inhibitor to enter clinical trials for prostate cancer, indicated a favorable tolerability profile as either monotherapy or in combination with taxane in four heavily pre-treated prostate cancer patients (144). Clinical studies are warranted to evaluate the clinical utility of FASN inhibitors in mCRPC.

Moreover, several drugs that target other enzymes in the *de novo* lipogenesis pathway are in clinical trials for other diseases, such as the ACC inhibitors Firsocostat (Gilead) and PF-05175157 (Pfizer) for non-alcoholic fatty liver disease (145), and derivatives of these compounds could conceivably be adopted for treatment of prostate cancer. Indeed, PF-05175157 showed promising results in reducing proliferation and inducing apoptosis in localized prostate cancer patient-derived explants (88).

### Fatty Acid Oxidation Inhibitors

Etomoxir is an irreversible inhibitor of carnitine palmitoyl transferase 1, which is the protein that transports fatty acids into the mitochondria for eventual oxidation. Treating mice with etomoxir reduced tumor growth in VCaP xenografts, without changing body weight or inducing systemic toxicity (67); however, etomoxir caused hepatotoxicity in patients with heart failure leading to the premature termination of a phase II clinical trial (146). While etomoxir is unlikely to progress to clinical trials for prostate cancer, two angina medications, ranolazine and perhexiline, may prove to be efficacious. Ranolazine is an FDA-approved partial inhibitor of fatty acid oxidation (147), while perhexiline is an TGA-approved competitive inhibitor of CPT1 (148). While neither drug reduces tumor growth alone, combining either compound with enzalutamide significantly decreased tumor growth *in vitro* and *in vivo* (149). Moreover, perhexiline alone showed no anti-tumorigenic activity in patient-derived explants, while cotreatment of perhexiline with the HSP90 inhibitor, AUY922, significantly reduced proliferation and increased apoptosis (150). These observations indicate that inhibitors of fatty acid oxidation may sensitize prostate cancer to other therapies, albeit through unknown mechanisms, and could be rapidly translated to the clinic.

### Glutaminolysis Inhibitors

CB-839, an oral glutaminase inhibitor, showed encouraging safety and tolerability results in a phase 1 study conducted in patients with advanced and/or treatment-refractory solid tumors, including breast cancer, lung cancer, renal cell carcinoma and mesothelioma (151). Preclinical studies in DU145 cells and xenografts indicated a synergistic effect of CB-839 in combination with talazoparib (PARP inhibitor) (152), leading to an upcoming phase II open label study of CB-839 and talazoparib in patients with mCRPC (NCT04824937).

### HMG-CoA Reductase Inhibitors

Statins are a class of drugs that inhibit the activity of HMG-CoA reductase and are widely used to treat patients with hypercholesterolemia. While observational studies demonstrate that statin use is associated with reduced cancer-specific mortality in patients with mCRPC receiving ADT (153), the results from one randomized trial indicates that short-term statin use does not impact tumor proliferation or serum prostate-specific antigen (PSA) compared to placebo (154). Similarly, statins alone did not reduce tumor burden in LNCaP xenograft and PDX trials; however, combination therapy with a re-purposed SREBP2 inhibitor, dipyridamole, significantly reduced tumor growth (155). Future studies exploring the safety and efficacy of this, and other combinations, in clinical studies are yet to be seen.

### Metformin

Metformin is the current first-line treatment of type 2 diabetes. While the exact mechanisms of action of metformin are still incompletely resolved, the anticancer potential of metformin is indicated through the capacity to activate AMPK and inhibit the cell cycle and epithelial-mesenchymal transition [*as reviewed in* (156)]. However, epidemiology studies showed no effects in reducing prostate cancer incidence and minimal improvement in overall survival (157). Multiple clinical trials are currently underway to assess the therapeutic utility of metformin as a monotherapy, or in combination with androgen targeted agents (enzalutamide and abiraterone) in managing CRPC.

## Prospects and Challenges for Implementing Metabolic Therapies in Prostate Cancer

Prostate cancer is slow growing by nature, providing sufficient time to implement therapies to delay progression or manage aggressive disease. For patients with intermediate risk disease, the median time to biochemical recurrence is ~4.25 years (158), necessitating the need for initiation of ADT, and in some patients, radiotherapy. Current clinical practice is to combine ADT with androgen-targeted therapy or chemotherapy, as this approach has been shown to increase overall survival (159). While effective in the short term, CPRC inevitably develops in ~5-8 years (160), which is then associated with a median survival ranging from 13-30 months (161–163). Overall, the time from diagnosis to end-stage disease for most patients is ~10-15 years, providing ample time for therapeutic intervention (**Figure 1**). This makes prostate cancer distinct to other more rapidly progressing cancers.

Of course, the overarching challenge in developing and utilizing ‘metabolic therapies’ for prostate cancer is to determine the appropriate strategy for the appropriate patient at the appropriate time, which as outlined above will vary between localized, metastatic and CRPC (see *Prostate Cancer Metabolism* section). We are, however, some way off implementing precise, actionable therapies as the focus of current research in cancer metabolism is predominantly pre-clinical and there is an urgent need for clinically based metabolic research. One emerging methodology, not yet applied to prostate cancer, is the use of intraoperative ^13^C metabolic tracer infusions in human cancer patients, which overcomes limitations of *ex vivo* studies and by integrating systemic, TME and spatial parameters that shape metabolic phenotypes (164).

The clinical trajectory described above is generalized for patients with intermediate-risk prostate cancer, although in reality, each patient has individual prognostic features that dictate disease progression. Risk-stratification for prostate cancer is critical to guide appropriate treatment decision-making. Towards this, it is worth considering whether there are subsets of patients who might benefit from metabolic therapies, either based on the reliance of an essential metabolic substrate, or specific tumor subtypes with common genomic aberrations or pathology. However, this has not been demonstrated, likely because of the remarkable heterogeneity of prostate cancer, diversity in metabolic substrate fluxes described in human tumors, and lack of appropriate biomarkers. In this context, mass spectrometry metabolic imaging is being refined to detect ‘metabolic signatures’ of prostate cancer, with evidence indicating that such imaging may aid in understanding biological processes and to help cancer diagnosis, prognosis and monitor response to therapies (165, 166).

A major challenge for the field is to define when metabolic therapies could be clinically applied. One option is during early-stage disease, following curative intent surgery or radiation when PSA levels are beginning to slowly rise, indicative of residual disease that is progressing. It is envisaged that metabolic therapies designed to reduce nutrient supply and/or ATP production could slow growth and delay the need for ADT. Alternatively, there is interest in the potential for metabolic therapies to be used to treat CRPC, because significant energy is required for the growth of highly aggressive therapy resistant tumors (**Figure 1**). More generally, it has been suggested that a better understanding of the association between metabolism and prostate cancer may lead to cancer prevention, although such strategies are opaque.

Overall, there is very little evidence from preclinical models or clinical studies that targeting a single metabolic pathway will be sufficient to slow prostate tumor progression. Firstly, this requires modulation of a single substrate, enzyme or metabolic pathway to limit tumor growth or increase tumor susceptibility to an adjunct therapy. In this context, metabolic inhibition, commonly leads to compensatory upregulation of other fuel utilization pathways to maintain pro-tumorigenic energy demands. For example, our work showed that this was the case with fatty acid transport inhibition, whereby blocking FAT/CD36 induced an increase in *de novo* lipogenesis in localized disease (38). Similarly, others showed that inhibition of FASN led to the upregulation of genes involved in steroid biosynthesis and increased intracellular cholesterol (143, 167). Thus, we posit that targeting dual processes will most likely be required for effective metabolic intervention in prostate cancer. Further to this, most tissues in the body readily utilize each of the substrates commonly used in prostate cancer, with evidence of dependencies in some tissues (*e.g.* glucose for red blood cells and brain). Hence, approaches that direct metabolic therapies to the tumor will be essential to minimize the likelihood of off-target effects. Such approaches are feasible as evidenced by the implementation of radioligand-therapy targeted to prostate-specific antigens in the clinic.

## Conclusions

Prostate cancer invokes major shifts in gene transcription and metabolic signaling to mediate alterations in nutrient acquisition and metabolic substrate selection when compared to normal tissues. Exploiting such metabolic reprogramming is proposed to enable the development of targeted therapies for prostate cancer, yet there are several challenges to be overcome before this becomes a reality. Firstly, several metabolic substrates have been identified in prostate cancer, including (but not limited to) fatty acids, glucose, lactate and glutamine, all of which are ‘required’ substrates in prostate cancer. Thus, identifying the most appropriate substrate to be targeted, and in which type of prostate cancer, remains unclear. Somewhat related, there is a gap in our knowledge of metabolism in human tumors. The majority of studies that have defined metabolic regulation of prostate cancer have been limited to cell culture or genetically modified mouse models, which does not accurately reflect the complexity of the *in vivo* tumor microenvironment and the impact that this induces on prostate metabolism (**Table 1**). Thirdly, prostate cancer is notoriously heterogeneous and there is currently insufficient evidence to indicate that subgroups of patients or tumor subtypes, based on genomic aberrations or pathology, share common metabolic vulnerabilities. Hence, there is an urgent need for these gaps to be addressed before metabolic therapies can be designed and incorporated into clinical practice.

## Author Contributions

All authors (GF, MW, and RT) conceived the idea for the review, searched the literature, drafted the manuscript and provided critical revision of the manuscript for intellectual content. GF generated figures. MW and RT obtained funding and provided supervision. All authors contributed to the article and approved the submitted version.

## Funding

This work was supported by the Prostate Cancer Foundation of Australia (ID: PCFA–NCG 3313, awarded to MW, RT), the Diabetes Australia Research Trust (awarded to MW) and the Cancer Council of Victoria (APP1160217, awarded to MW, RT). MW was supported by the National Health and Medical Research Council NHMRC of Australia (ID: APP1077703), and RT by the Victorian Cancer Agency (MCRF15023). GF was supported by the Melbourne Research Scholarship (University of Melbourne).

## Conflict of Interest

The authors declare that the research was conducted in the absence of any commercial or financial relationships that could be construed as a potential conflict of interest.

## Publisher’s Note

All claims expressed in this article are solely those of the authors and do not necessarily represent those of their affiliated organizations, or those of the publisher, the editors and the reviewers. Any product that may be evaluated in this article, or claim that may be made by its manufacturer, is not guaranteed or endorsed by the publisher.
